# Comparative Analysis of Apoptotic Resistance of Mesenchymal Stem Cells Isolated from Human Bone Marrow and Adipose Tissue

**DOI:** 10.1100/2012/105698

**Published:** 2012-04-24

**Authors:** Gökhan Ertaş, Ertan Ural, Dilek Ural, Ayça Aksoy, Güliz Kozdağ, Gülçin Gacar, Erdal Karaöz

**Affiliations:** ^1^Department of Cardiology, Faculty of Medicine, Bezmialem Vakif University, Adnan Menderes Bulvarı Vatan Caddesi, Fatih, 34093 Istanbul, Turkey; ^2^Department of Cardiology, Faculty of Medicine, Kocaeli University, 41380 Kocaeli, Turkey; ^3^Stem Cell and Gene Therapy Research and Applied Center, Faculty of Medicine, Kocaeli University, 41380 Kocaeli, Turkey

## Abstract

*Aim*. Mesenchymal stem cells (MSCs) isolated from human bone marrow (hBM) and adipose tissue (hAT) are perceived as attractive sources of stem cells for cell therapy. The aim of this study was to compare MSCs from hBM and hAT for their immunocytochemistry staining and resistance to in vitro apoptosis. *Methods*. In our study, we investigated the antiapoptotic ability of these MSCs toward oxidative stress induced by hydrogen peroxide (H_2_O_2_) and serum deprivation. Results were assessed by MTT and flow cytometry. All experiments were repeated a minimum of three times. *Results*. Flow cytometry and MTT analysis revealed that hAT-MSCs exhibited a higher resistance toward H_2_O_2_-induced apoptosis (*n* = 3, hBM-hAT viability H_2_O_2_  58.43 ± 1.24–73.02 ± 1.44, *P* < 0.02) and to serum-deprivation-induced apoptosis at days 1 and 4 than the hBM-MSCs (*n* = 3, hAT-hBM absorbance, resp., day 1: 0.305 ± 0.027–0.234 ± 0.015, *P* = 0.029, day 4: 0.355 ± 0.003–0.318 ± 0.007, *P* = 0.001, and day 7: 0.400 ± 0.017–0.356 ± 0.008, *P* = 0.672). hAT-MSCs showed superior tolerance to oxidative stress triggered by 2 mmol/L H_2_O_2_ and also have superior antiapoptosis capacity toward serum-free culture. *Conclusion*. In this study we found that hAT-MSCs are more resistant to in vitro apoptosis.

## 1. Introduction

Ischaemic heart disease (IHD) is the major cause of congestive heart failure and subsequent mortality in developed countries. Despite the most advanced therapeutic and interventional treatments, such as percutaneous coronary intervention (PCI) or coronary artery bypass graft (CABG), the number of patients suffering from heart failure is increasing. End-stage disease can only be treated by heart transplantation when all other medical treatments have failed. But it is hampered by a lack of suitable donor organs [1]. Cell transplantation has raised hope as a new therapeutic modality. Cell transplantation as an adjunctive therapy to promote myocardial function after an acute myocardial infarction (AMI) has been widely studied in both experimental and clinical conditions. Results from studies have revealed that injection of stem cells can improve myocardial function by inducing angiogenesis and inhibiting apoptosis [2, 3]. The goal of cell transplantation is the substitution of scarred myocardium with viable cells ultimately leading to overall improvement of myocardial function. Two of the most widely used cell types for cardiac repair today are myoblasts and human-bone-marrow- (hBM-) derived progenitors. Human adipose tissue (hAT) is also perceived as attractive source of stem cells for cell therapy. hAT-derived cells in the infarcted heart have never been compared directly to hBM-derived mesenchymal stem cells (MSCs) in clinical trials. Although the in vitro properties of hAT- and hBM-derived MSCs have been compared before [4, 5], there are no reports evaluating antiapoptotic ability of MSCs isolated from hBM and hAT. The aim of this study was to compare MSCs from hBM and hAT for their immunocytochemistry staining and resistance to in vitro apoptosis.

## 2. Materials and Methods

All of the biological materials used in this study were collected after the approval of the Ethics Committee, Kocaeli University, and informed consent was signed by the patients according to institutional guidelines under the approved protocol.

### 2.1. Isolation and Culture of Human BM-Derived MSCs

hBM-MSCs were isolated from bone marrow of the patients who went under immune thrombocytopenic purpura. Mononuclear cells were separated by centrifugation over a Ficoll-Hypaque gradient (Sigma Chemical Co., St. Louis, MO) and suspended in *α*-MEM medium (Biochrom-FG0325) containing 15% FBS (FBS; Invitrogen/GIBCO, Grand Island, NY, USA) and 100 IU/mL penicillin-100 *μ*g/mL streptomycin (Invitrogen/GIBCO) followed by plating at an initial seeding density of 1 × 10^6^ cells/cm^2^. All of the cells isolated from five samples were plated in different 25 cm^2^ medium-containing culture flasks. After seven days of incubation, the media were replaced, and replacement was then performed twice a week. In the primary cell culture after cells reached confluency of 80–90% they were treated with 0.025% trypsin-EDTA for 3 min, and the released cells were collected by centrifugation and replated at a rate of 1 : 3-1 : 4 for subculturing. Passage 3 MSCs were used in all experiments.

### 2.2. Isolation and Culture of Human AT-Derived MSCs

In brief hATs were obtained from subcutaneous material after uncomplicated elective caesarean deliveries from healthy mothers. Tissue samples were washed several times with Hanks' balanced salt solution (HBSS) with 5% antibiotic-antimycotic solution and without calcium and magnesium to remove blood (Invitrogen). Tissues were minced into small blocks and a single cell suspension of adipose tissue cells was obtained by using enzymatic digestion and mechanical means.

The enzymatic digestion procedure was performed as described below.

The finely cut hATs were moved to a 50 mL conical tube (BD Biosciences) and then chemically decomposed in *α*-MEM (Modified Eagle Medium, Gibco) medium supplemented with 5 mL of %0,075′lik collagenase type 2 (SIGMA, St. Louis, MO) at 37°C for 60 minutes in a shaking water bath rotating at 150 rpm. At 20 min intervals, the digests were pipetted vigorously and dissociation monitored microscopically. After approximately 60 minutes the cell suspensions were filtered using a 70 *μ*m cell strainer (Becton Dickinson Labware, Franklin Lakes, NJ, USA) to separate single cells from debris and undigested adipose tissue fragments. Cells were seeded into 25 cm^2^ culture flask containing *α*-MEM supplemented with 100 U/mL penicillin, 0.1 mg/mL streptomycin, and 15% FBS. Seven days after the initiation of culture, the medium was changed twice a week. After cells reached 80–90% confluence, they were treated with 0.025% trypsin-EDTA for 3 min. The released cells were collected, centrifuged, and replated at rate of 1 : 3-1 : 4 for subculture.

## 3. Flow Cytometry

To confirm that MSCs maintain their phenotypic characteristics after growth in culture, undifferentiated SCs were subjected to flow cytometry analysis. After each passage, stem cells were harvested and suspended in their own culture medium at a concentration of 1 × 10^6^ cells/mL. Flow cytometry was performed by using a FACS Calibur (BD Biosciences, San Diego, USA). The data were analysed with Cell Quest software (BD Biosciences) and the forward and side scatter profile gated out debris and dead cells.

Immunophenotyping of MSCs was performed with antibodies against the following human antigens: CD3, CD8, CD10, CD11b, CD13, CD14, CD15, CD19, CD33, CD34, CD44, CD45, CD71, CD73, CD90, CD117, CD146, CD166, and HLA-DR. All of the antibodies were supplied by Becton Dickinson.

## 4. Immunohistochemistry

To identify cellular markers, P3 cells were seeded on poly-L-lysine-coated 8-well chamber slides (BD Biosciences), cultured for another 1-2 days and subjected to immunocytochemistry and immunofluorescence staining.

### 4.1. Immunofluorescence Staining

Samples were rinsed briefly in PBS, fixed in ice-cold methanol for 10 min, and then allowed to dry completely. After permeabilisation with 0.025% Triton X-100 (Merck, Darmstadt, Germany), the cells were incubated with 1.5% normal goat or donkey blocking serum (Santa Cruz Biotechnology) in PBS for 30 min at 37°C to suppress nonspecific binding of IgGs. After washing three times with PBS (5 min each) the cells were incubated overnight at 4°C with the primary antibodies listed in Table 1. After three PBS washes, cells were incubated with FITC and Texas-red- (Santa Cruz Biotechnology) labelled appropriate secondary antibodies for 25 min in dark. After washing three times with PBS, the cells were mounted with mounting medium containing DAPI (Santa Cruz Biotechnology).

### 4.2. Immunoperoxidase Staining

Immunocytochemical analysis was performed using the streptavidin-peroxidase method (UltraVision Plus Large Volume Detection System Anti-Polyvalent, HRP immunostaining Kit, Thermo Scientific, UK). To reduce nonspecific background staining due to endogenous peroxidase, cultured cells were fixed in ice-cold methanol with 0.3% hydrogen peroxide (Carlo Erba Reactifs, Val-De-Reuil Cedex Pa Des Portes, FRANCE) for 15 min and allowed to dry. After additional PBS washes, cells were incubated with Ultra V Block for 5 min at room temperature. Then, cells were incubated overnight at 4°C with the primary antibodies listed in Table 1. The following day, cells were incubated with biotinylated secondary antibodies for 15 min at room temperature. Incubations were followed by streptavidin peroxidase treatment for 15 min at room temperature and signals were detected with the AEC kit (Zymed Laboratories, UK). The cells were counterstained with hematoxylin (Santa Cruz Biotechnology) and examined under a light microscope (Leica DMI 4000B, Wetzlar, Germany). After induction of apoptosis (described below) cells were stained with caspase-3.

## 5. Apoptosis Induction and Detection

### 5.1. 2 mmol/L H_2_O_2_-Induced Apoptosis

At passage 3 hBM-MSCs and hAT-MSCs (*n* = 3) were seeded into 6-well plate at a density of 10 × 10^4^/cm^2^ and cultured for a further 48 h and medium changed to apoptosis inducing medium that contains 2 mmol/L H_2_O_2_ MEM ve 10% FBS. After 60 min, cells were washed with PBS and apoptotic cell percentage was detected by flow cytometry with Annexin-V-FITC Apoptosis Detection Kit (BD Pharmingen). The data were analyzed with the Cell Quest software (BD Biosciences).

### 5.2. Serum-Deprivation-Induced Apoptosis

Analysis of MTT was made using MTT Cell Growth Kit (Chemicon). hAT- and hBM-MSCs at passage 3 were seeded at 12.500 cells per well in 6-well plates and were incubated in 5% CO_2_, 37°C for 1, 4, and 7 days. Then culture medium was replaced. Wells were washed twice with phosphate-buffer saline (PBS). In the growth curve experiment, 10 *μ*L MTT (0.5 mg/mL) was added and the culture was incubated for 4 h. 100 *μ*L isopropanol/HCl was added to culture medium. Absorbance at 570 nm was measured by a UV-visible spectrophotometer microplate reader (VersaMax, Molecular Device, USA). For each group, experiments were repeated three times, and measurements were done in triplicates.

### 5.3. Statistical Analysis

 All experiments were performed as triplicates. Data are reported as means ± SD. All statistical analyses were performed using SPSS 10.0 (SPSS Inc., Chicago, IL, USA). Data were analyzed using one-way ANOVA and paired *t*-test. Differences between the experimental and control groups were regarded as statistically significant when *P* < 0.05.

## 6. Results

### 6.1. Isolation and Culture of hBM-MSCs and hAT-MSCs

**Figure 1:**
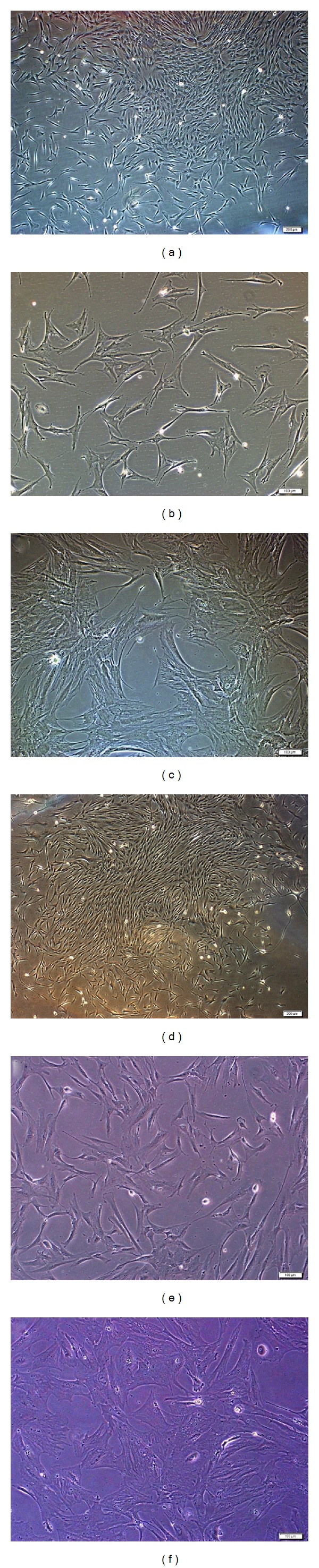
Representative fields showing hBM- and hAT-MSCs morphologies for different passages. (a-b) hBM-MSCs in culture. During the onset of culture P_0_: 12th day (a), P_2_: 1st day (b), and P_3_: 7th day (c). MSCs attached to the culture flasks sparsely and displayed a fibroblast-like, spindle-shaped morphology during the initial days of incubation. After 5–7 days of incubation, proliferation started and the cells gradually grew into small colonies (a). After the next passages, most of these MSCs exhibited large, flattened morphology (b, c). (c-d) Representative fields showing hAT-MSCs morphologies for different passages ((c) P_0_: 12th day, (d) P_2_: 4th day, and (e) P_3_: 6th day). hAT-MSCs were observed to appear morphologically very similar to hBM-MSCs.

MSCs attached to the culture flasks sparsely and displayed a fibroblast-like, spindle-shaped morphology during the initial days of incubation. After 3-4 days of incubation, proliferation started and the cells gradually grew into small colonies named colony-forming units (CFU). By the time they are 6 to 8 days of age, colonies with different sizes increased in number. As growth continued, adjacent colonies interconnected with each other and a monolayer confluence was obtained after 12 to 16 days of incubation (Figures 1(a) and 1(d)). In later passages, MSCs exhibited large, flattened or fibroblast-like morphology (Figures 1(b), 1(c), 1(e), and 1(f)).

### 6.2. Flow Cytometry Identification of hAT- and hBM-MSCs

**Figure 2:**
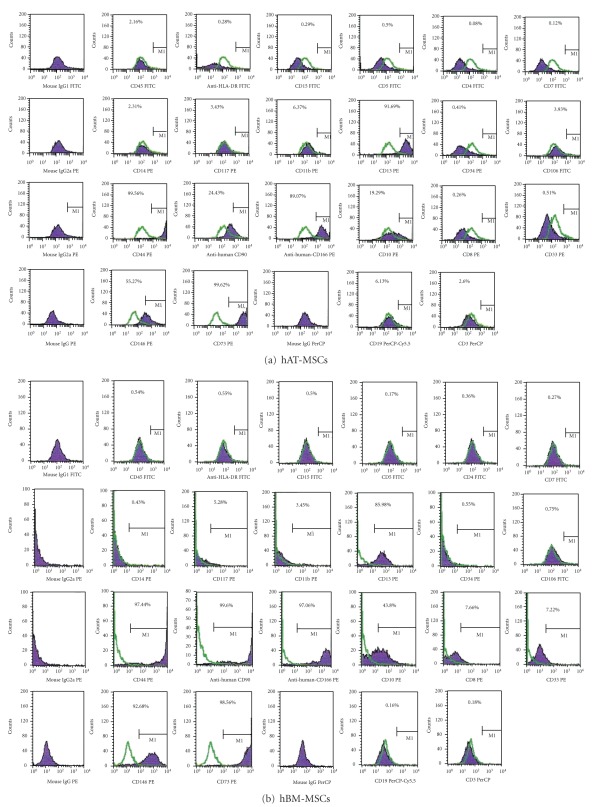
Representative flow cytometry analysis of cell-surface markers in hAT-MSCs (a) and hBM-MSCs (b) at passage 3. Cells were labeled with antibodies against hematopoietic antigens (CD3, CD8, CD10, CD14, CD15, CD33, CD34, CD45, CD71, CD117, and HLA-DR) and MSC markers (CD13, CD44, CD73, CD90, CD146, and CD166) or immunoglobulin isotype antibodies and analyzed by flow cytometry as above. Green line, control immunoglobulin. A representative example of more than two experiments.

 Defined markers exist that especially and uniquely identify MSCs. We utilised some markers to define our cultured cells. Our data indicated that hAT-MSCs and hBM-MSCs expressed CD13, CD44, CD73, CD90, CD146, and CD166, but not CD3, CD8, CD10, CD11b, CD14, CD15, CD19, CD33, CD34, CD45, CD71, CD117, and HLA-DR. These findings are consistent with their undifferentiated state, and, similar to hBM-MSCs, they possessed immunophenotypic MSCs characteristics as shown in Figures 2(a) and 2(b).

### 6.3. Immunocytochemical Properties of hAT- and hBM-MSCs

**Figure 3:**
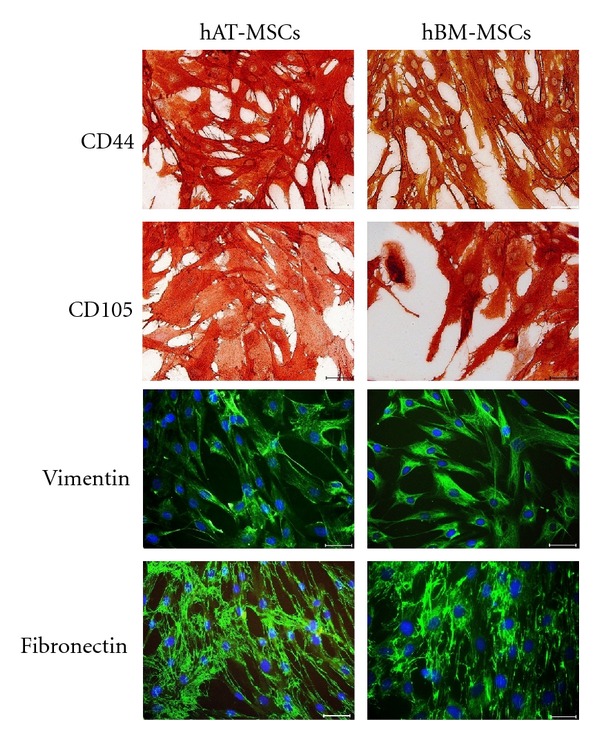
Immunophenotype of cultured hAT- and hBM-MSCs. Studies based on immunoperoxidase and immunofluorescence reactivities were performed on third passage cultures of hMSCs. Representative staining patterns are shown for CD 44, CD105, vimentin, and fibronectin. Nuclei were counterstained with haematoxylin and were labeled with DAPI (blue). Scale bars = 50 *μ*m.

Typical immunoreactivity profiles of hAT- and hBM-MSCs are specified in Table 1. Under the standard culture conditions, these cells expressed MSC markers such as CD105/endoglin, CD44, CD146, vimentin, and fibronectin and their morphological characteristics remained unchanged (Figures 2 and 3). As summarized in Table 1, hAT- and hBM-MSCs did not express surface markers like CD31, CD34, and CD45 (hematopoietic markers) or CD71 (transferrin receptor).

### 6.4. Antiapoptosis Ability of MSCs

**Figure 4:**
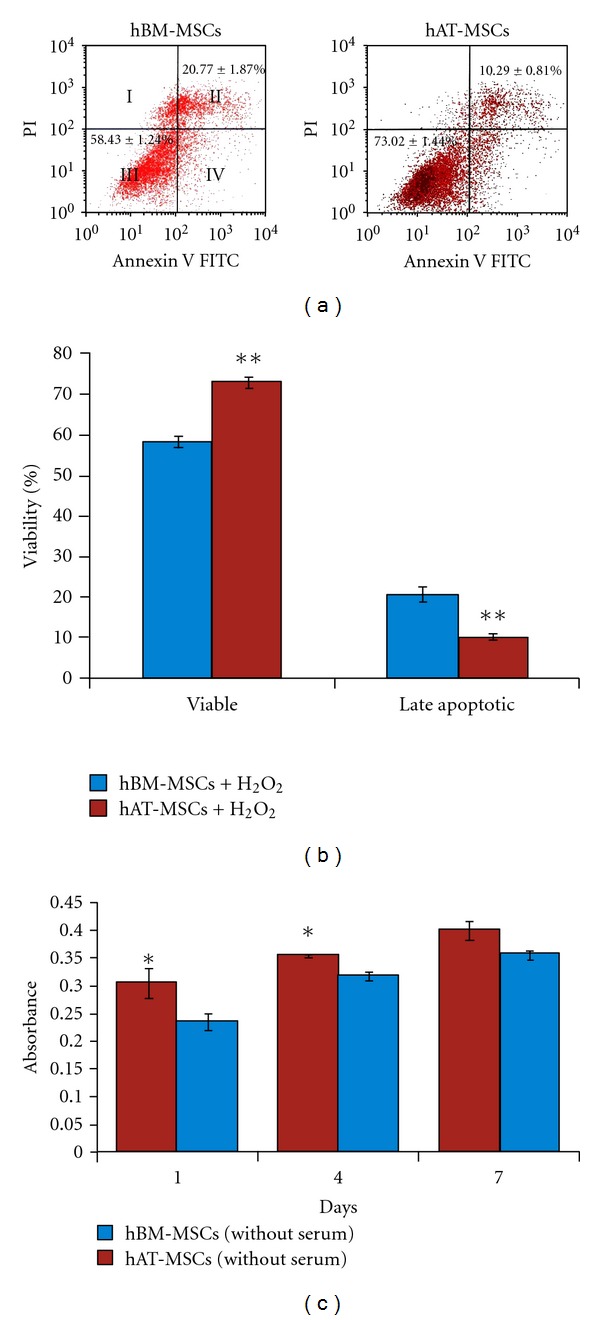
(a-b) Apoptosis was quantified by FACS analysis after staining with Annexin V and PI after incubation with H_2_O_2_ for 60 min. Cell apoptosis was measured by Annexin V-FITC, which binds to phosphatidylserine residues that are redistributed from the inner to the outer leaflet of the cell membrane as an early event in apoptosis. After loss of membrane integrity, PI can enter the cell and intercalate into DNA (I: necrotic cell; II: late apoptotic cell; III: viable cell; IV: early apoptotic cell.) (c) Serum deprivation induced a reduction in viability determined by MTT. The results were presented as the ratio of apoptosis induced by 2 mmol/L H_2_O_2_ or serum deprivation (mean ± SD, *n* = 3 each, *P < 0.05, **P < 0.01).

 Apoptosis triggered by 2 mmol/L H_2_O_2_. After 60 min of 2 mmol/L H_2_O_2_ induction, obvious morphology changes in the hAT- and hBM-MSCs were observed by light microscopy (data not shown). Cell apoptosis was measured by Annexin V-FITC, which binds to phosphatidylserine residues that are redistributed from the inner to the outer leaflet of the cell membrane as an early event in apoptosis. After loss of membrane integrity, PI can enter the cell and intercalate into DNA [6]. Figures 4(a) and 4(b) show the percentages of Annexin V-PI-stained cells of hAT- and hBM-MSCs. The average percentages of Annexin V+-PI+ (late apoptotic cells) were the highest in hBM-MSCs (20.77%  ± 1.87), and the percentage in hBM-MSCs was the lowest (10.29%  ± 0.81). As shown in Figures 4(a) and 4(b), H_2_O_2_ induced a significant decrease in the viability rates of hAT-MSCs compared with hBM-MSCs (73.02 ± 1.44–58.43 ± 1.24, resp., *P* = 0.002). Moreover, there was a statistical significance between hAT-MSCs and hBM-MSCs as well as the rate of Annexin V+/PI− (early apoptotic cells) and Annexin V−/PI+ (necrotic cells) (data not shown). Therefore, this suggested that hAT-MSCs had a superior tolerance to H_2_O_2_-induced cytotoxicity.

During ischemia, multiple changes contribute to cellular death. Among these are deprivation of nutrients, growth and survival factors, and oxygen. Serum deprivation is known to induce apoptosis in various cell types including stem cells [7]. To determine whether MSCs can tolerate ischemia, the cells were exposed to serum deprivation (SD). After serum withdrawal for 1, 4, and 7 days, hAT- and hBM-MSCs were analyzed by MTT. Active mitochondrial dehydrogenase of living cells can cleave MTT to produce formazan, the amount of which directly correlates with the number of metabolically active cells. hBM-MSCs showed inferior tolerance to serum-free culture than hAT-MSCs. In serum-free culture, proliferation index of hAT-MSCs that has shown viable cell ratio was higher than that of hBM-MSCs at the 1st and 4th days (*n* = 3, hAT- and hBM-MSCs mean absorbance values, resp., for the 1st day: 0.305 ± 0.027, 0.236 ± 0.015, *P* = 0.042, for the 4th day: 0.355 ± 0.003, 0.318 ± 0.007, *P* = 0.011). Interestingly, there was not a statistically significant difference between the survivability of MSCs at the 7th day (*n* = 3, hAT- and hBM-MSCs mean absorbance values, resp., the 7th day: 0.400 ± 0.017, 0.356 ± 0.008, *P* = 0.081) (Figure 4(c)).

 Caspase-3 is the most extensively studied apoptotic protein. Caspase-3 is synthesized as an inactive proenzyme that is processed in cells undergoing apoptosis by self-proteolysis and/or cleavage by another upstream protease. The caspase-3 immunohistochemistry technique is a simple, easy, and reliable method for the early identification and quantification of apoptotic cells in histological sections [8]. hBM-MSCs showed the most sensitive reaction to oxidative stress in that most of cells showed intense caspase-3 immunoreactivity (data not shown). In contrast to hBM-MSCs, hAT-MSCs showed superior tolerance to oxidative stress with the least morphological change.

## 7. Discussion

MSCs are showing great potential for the treatment of cardiovascular diseases, in particular ischaemic heart disease and heart failure. Results from animal studies and initial human trials are encouraging [9–17]. MSCs can be found in bone marrow, muscle, skin, dental pulp, cord blood, amniotic fluid, and adipose tissue. MSCs are characterized by the potential to differentiate into muscle, fibroblasts, bone, and adipose tissue [18]. Two of the most widely used cell types for cardiac repair today are myoblasts and BM-derived progenitors. Adipose tissue (AT) is also perceived as attractive source of stem cells for cell therapy. To our knowledge this study is the first study comparing antiapoptotic ability of hBM-derived MSCs and hAT for the purpose of setting up an in vitro evaluation test to help choosing a better cell source for clinical trials.

The major findings are as follows: (1) hAT-MSCs are a promising source due to their high proliferation ability. (2) Flow cytometry and MTT analysis showed that hAT-MSCs possess higher resistance toward H_2_O_2_- and serum-deprivation-induced apoptosis than hBM-MSCs.

Kern et al. compared morphology, the success rate of isolating MSCs, colony frequency, expansion potential, multiple differentiation capacity, and immune phenotype of human MSCs isolated from umbilical cord blood (UCB), BM, and AT. This study revealed that the success rate of isolating MSCs was higher in AT and BM than in UCB. Unlike AT, which had the highest colony-forming frequency, the colony-forming frequency was the lowest in UCB. UCB-MSCs showed no adipogenic differentiation capacity, in contrast to BM- and AT-MSCs. Authors concluded that UCB and AT are attractive alternatives to BM in isolating MSCs [19].

Peng et al. compared immunophenotypic characterization, proliferation capacity, differentiation ability, expression of angiogenic cytokines, and antiapoptotic ability of MSCs of adult Sprague Dawley rats derived from adipose tissue, bone marrow, and cartilage [7]. They investigated the antiapoptotic ability of these MSCs toward oxidative stress induced by H_2_O_2_ and serum deprivation. In this study, based on the growth curve, cell cycle, and telomerase activity analyses, MSCs derived from AT-MSCs possess the highest proliferation potential, followed by MSCs derived from bone marrow and cartilage. In terms of multilineage differentiation, MSCs from all three sources have displayed osteogenic, adipogenic, and chondrogenic differentiation potential. Flow cytometry and MTT analysis have showed that cartilage-MSCs possess the highest resistance toward H_2_O_2_-induced apoptosis, while AT-MSCs have exhibited high tolerance to serum-deprivation-induced apoptosis. Authors concluded that adipose tissue and cartilage are attractive alternatives to bone marrow as sources for isolating MSCs [7].

To our knowledge there is only a single in vivo study comparing fate and function of adipose stromal cells (ASCs) in the infarcted heart directly to BM-derived MSCs. ASCs and MSCs were isolated from transgenic FVB mice. Unfortunately, the authors concluded that ASCs and MSCs did not tolerate well in the cardiac environment, resulting in acute donor cell death and a subsequent loss of cardiac function similar to control groups [20]. hAT-derived MSCs have not yet been compared directly to hBM-derived MSCs in clinical studies.

The results of this study suggest that hAT may represent an ideal source of autologous stem cells for stem cell treatment. However, the process of bone marrow harvesting can be painful and quantity of cells that can be harvested from the patient is limited. Liposuction may be a good choice for adipose tissue harvesting. hAT-derived MSCs largely express the same surface markers as BM-MSC and have shown to preserve cardiac function after infarction [21].

## 8. Conclusion

The ability to resist stress and apoptosis is an important phenomenon for survival. This is especially important in stem cell studies during which stem cells are being injected to a damaged area for recovery purposes. If a stem cell is more prone to apoptosis, the change of survival during clinical practice would be less. Therefore, stem cells selected for clinical studies should be checked for their survival rate under stres conditions. In this study we found that hAT-MSCs are more resistant to in vitro apoptosis.

## Figures and Tables

**Table 1:** Immunocytochemical properties of hAT-MSCs and hBM-MSCs.

Antibody/marker	Dilution	Cell type	
		hAT-MSCs	hBM-MSCs
		detection	detection
CD 31/PECAM-1 (M-20)	1 : 100	⌀	⌀
CD 34 (C-18)	1 : 150	⌀	⌀
CD 44/HCAM (Ab-4)	1 : 150	+	+
CD 45 (H-230)	1 : 150	⌀	⌀
CD 71 (K-20)	1 : 150	⌀	⌀
CD105/Endoglin (M-20)	1 : 100	+	+
Vimentin (C-20)	1 : 100	+	+
Fibronectin (EP5)	1 : 100	+	+
